# Neural processing in the primary auditory cortex following cholinergic lesions of the basal forebrain in ferrets

**DOI:** 10.1016/j.heares.2024.109025

**Published:** 2024-06

**Authors:** Fernando R. Nodal, Nicholas D. Leach, Peter Keating, Johannes C. Dahmen, Dylan Zhao, Andrew J. King, Victoria M. Bajo

**Affiliations:** a Department of Physiology, Anatomy and Genetics, University of Oxford, Parks Road, Oxford OX1 3PT, United Kingdom; b Red Thread Market Access. United Kingdom; c UCL Ear Institute, 332 Gray's Inn Road, London WC1X 8EE, United Kingdom

**Keywords:** Acetylcholine, Nucleus basalis, Virtual acoustic space, Auditory cortex, Spectral sensitivity, Monaural occlusion

## Abstract

Cortical acetylcholine (ACh) release has been linked to various cognitive functions, including perceptual learning. We have previously shown that cortical cholinergic innervation is necessary for accurate sound localization in ferrets, as well as for their ability to adapt with training to altered spatial cues. To explore whether these behavioral deficits are associated with changes in the response properties of cortical neurons, we recorded neural activity in the primary auditory cortex (A1) of anesthetized ferrets in which cholinergic inputs had been reduced by making bilateral injections of the immunotoxin ME20.4-SAP in the nucleus basalis (NB) prior to training the animals. The pattern of spontaneous activity of A1 units recorded in the ferrets with cholinergic lesions (NB ACh^–^) was similar to that in controls, although the proportion of burst-type units was significantly lower. Depletion of ACh also resulted in more synchronous activity in A1. No changes in thresholds, frequency tuning or in the distribution of characteristic frequencies were found in these animals. When tested with normal acoustic inputs, the spatial sensitivity of A1 neurons in the NB ACh^–^ ferrets and the distribution of their preferred interaural level differences also closely resembled those found in control animals, indicating that these properties had not been altered by sound localization training with one ear occluded. Simulating the animals’ previous experience with a virtual earplug in one ear reduced the contralateral preference of A1 units in both groups, but caused azimuth sensitivity to change in slightly different ways, which may reflect the modest adaptation observed in the NB ACh^–^ group. These results show that while ACh is required for behavioral adaptation to altered spatial cues, it is not required for maintenance of the spectral and spatial response properties of A1 neurons.

## Highlights

• Reduction of the cholinergic input from the nucleus basalis does not result in a change in the frequency tuning of cortical neurons under anesthesia.
• Reduction of the cholinergic input from the nucleus basalis results in a decrease in the proportion of busting neurons in the primary auditory cortex and increased neural synchrony.
• Reduction of the cholinergic input from the nucleus basalis does not result in a change in the spatial tuning of cortical neurons despite animals showing impairment in the localization of brief sounds and an inability to behaviourally adapt to a monaural occlusion.

## 1 Introduction

The cholinergic basal forebrain plays an essential role in cognition. The loss of basal forebrain cholinergic cells correlates with the severity of cognitive deterioration in neurodegenerative diseases, such as Alzheimer's or Parkinson's (Francis et al., 1999; Whitehouse et al., 1982). Indeed, most drugs available for Alzheimer's disease act through inhibition of acetylcholinesterase (Sharma, 2019).

Inputs from the cholinergic basal forebrain can induce changes in cortical responses that are related to arousal, attention, learning and memory encoding (Bear and Singer, 1986; Everitt and Robbins, 1997; Fine et al., 1997; Froemke et al., 2007; Goard and Dan, 2009; Hasselmo and McGaughy, 2004; Jasper and Tessier, 1971; Letzkus et al., 2011; Sarter et al., 2005; Voytko et al., 1994; Weinberger, 2003) and are engaged during active behaviors that alter perception (Eggermann et al., 2014; Fu et al., 2014; Hangya et al., 2015; Herrero et al., 2008; Nuñez et al., 2012; Parikh et al., 2007; Pinto et al., 2013). Stimulating the basal forebrain simultaneously with sensory input evokes a transient cholinergic release in sensory cortices (Pinto et al., 2013) that enhances thalamocortical signaling and reduces intracortical transmission (Herrero et al., 2008; Hsieh et al., 2000).

The organization of the basal forebrain into four distinct groups is common across mammals despite species-specific differences in the proportion of cholinergic cells in each group (e.g. Mesulam et al., 1983a, b; Saper, 1984), the topography of the cortical projections, or the density and distribution of the different cholinergic receptors across cortical areas, layers and cells (reviewed in Coppola and Disney, 2018; Záborszky et al., 2018). The changes triggered by the tonic release of ACh in the sensory cortices have been widely reported (reviewed in Edeline, 2012; Hasselmo and Sarter 2011), and recent progress has been made in undercovering the role of phasic cholinergic signaling associated with sensory stimulation (Sarter and Lustig, 2019; Zhu et al., 2023). However, how loss of cholinergic neurons affects basic response properties of sensory cortical neurons is still poorly understood.

The nucleus basalis (NB, cholinergic group Ch4) provides extensive inputs to the amygdala and neocortex (Mesulam et al. 1983a, b) including prefrontal, cingulate, somatosensory, auditory cortices (Záborszky et al., 2015; Chavez and Záborszky, 2017; Eggermann et al., 2014; Kim et al., 2016; Woolf, 1991) and visual cortex (Gombkoto et al., 2021). In ferrets, we have previously shown that NB provides most of the cholinergic input to the auditory cortex (Bajo et al., 2014) and that this input is required for normal sound localization behavior and experience-dependent plasticity (Leach et al., 2013). Removal of cholinergic inputs to the auditory cortex by bilateral injections of the immunotoxin ME20.4-SAP in NB reduced the accuracy with which ferrets localize brief sounds and prevented them from adaptively reweighting auditory localization cues with training in response to occlusion of one ear.

Here, we investigated the contribution of ACh to the spectral and spatial response properties of neurons in the primary auditory cortex (A1). We compared the spontaneous and driven activity of A1 neurons in a group of control ferrets and in animals in which the cholinergic input to the auditory cortex had been eliminated using a selective immunotoxin (ME20.4-SAP) injected into the NB and which had subsequently been tested for their ability to localize sound and to adapt to altered spatial cues. The control group comprised naïve untrained ferrets to avoid any learning-related changes in cortical responses that would be expected to occur in animals with intact cholinergic inputs. Our results show that the long-term loss of cholinergic input does not alter the spectral or spatial tuning of auditory cortical neurons. The lack of changes in A1 spatial response properties is consistent with the greatly reduced plasticity observed behaviorally in these animals.

## 2 Methods

Experiments were carried out in ten pigmented adult ferrets (*Mustela putorius furo*). Given the sexual dimorphism found in ferrets, with adult males usually more than double the body weight of females, and to avoid miscalculations in the brain stereotaxic coordinates, only females weighing between 700 and 800 g at the time of surgery were used. Experiments were conducted following the Society for Neuroscience policies on the use of animals in neuroscience research http://www.sfn.org/Advocacy/Policy-Positions/Policies-on-the-Use-of-Animals-and-Humans-in-Research. All experimental procedures were approved by the University of Oxford Animal Care and Ethical Review Committee and performed under license from the UK Home Office under the Animals Scientific Procedures Act (1986, 2012).

### 2.1 Experimental design

**Fig. 1:**
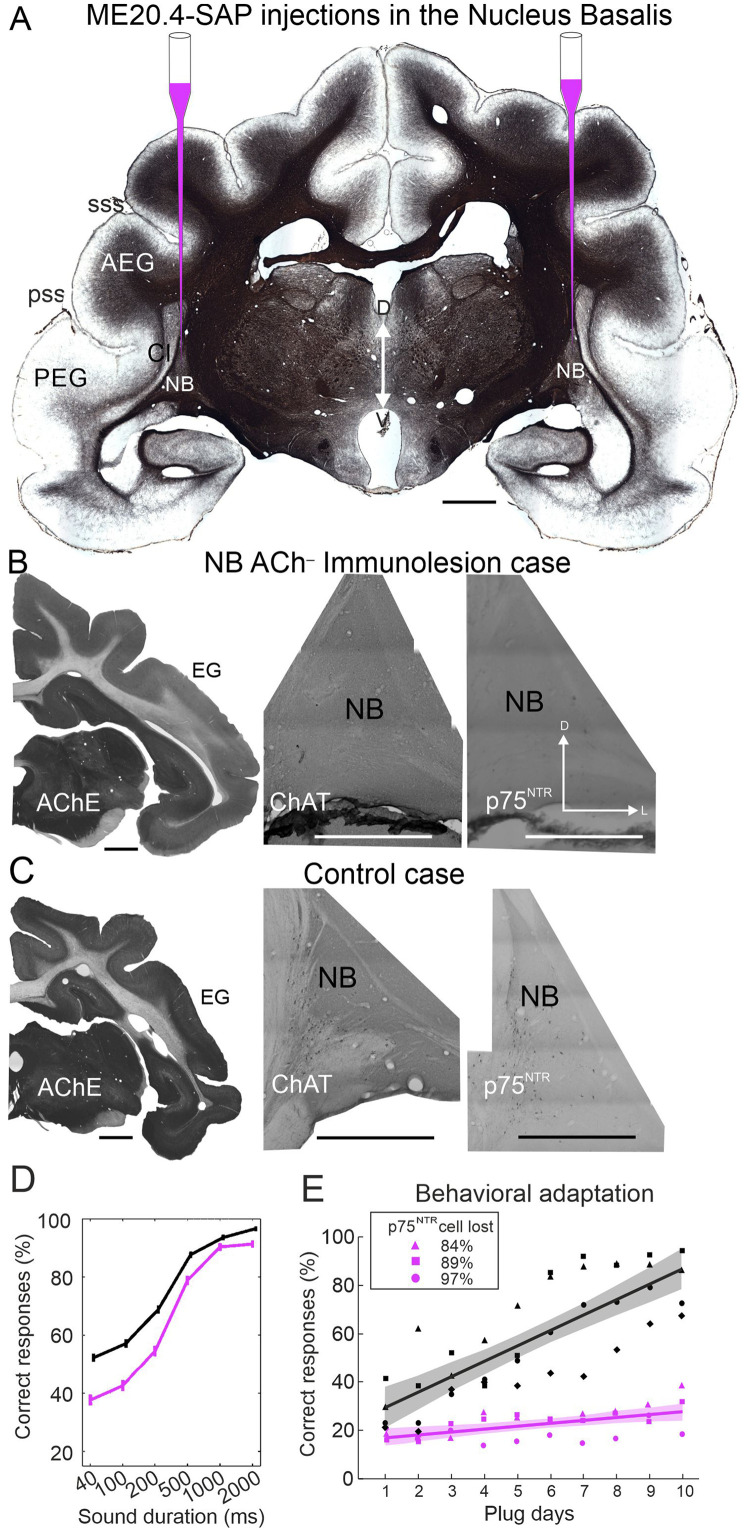
Experimental design and behavioral data.

We recorded spontaneous and sound-driven activity in A1 of anesthetized ferrets in two experimental groups. The control group (*n* = 7) consisted of untrained ferrets and in the second group (*n* = 3), we injected the immunotoxin ME20.4-SAP bilaterally into the NB to deplete cholinergic neurons (NB ACh^–^ group) (Fig. 1). The injections were made six months before the recordings were performed (Fig. 1A). NB ACh^–^ animals were then behaviorally tested on a sound localization task (Fig. 1D) and for their ability to adapt to the altered spatial cues caused by the monaural occlusion (Fig. 1E). Behavioral results from NB ACh^–^ ferrets and from ferrets without cholinergic lesions have been reported elsewhere (Leach et al., 2013; Sanchez Jimenez et al., 2023).

**A**, We aimed to remove cholinergic inputs to the auditory cortex by making bilateral injections of the immunotoxin ME20.4-SAP in the nucleus basalis (NB). Scale bar 2 mm. **B** and **C**, Pattern of acetylcholinesterase (AChE) staining in the ectosylvian gyrus (EG) where the auditory cortex is located (left panel, scale bar 2 mm) and the pattern of immunostaining in the NB for choline acetyltransferase (ChAT) (middle panel, scale bar 0.5 mm) and p75 neurotrophin receptor (p75^NTR^) (right panel, scale bar 0.5 mm). An example of an NB ACh^—^ immunolesion case (**B**) and an example of a control case (**C**). Only the right side of the brain is illustrated in **B** and **C**, although NB injections were bilateral. **D**, Effect of cortical cholinergic depletion on sound localization accuracy across different sound durations. **E**, Effect of cortical cholinergic depletion on behavioral adaptation to a unilateral left earplug in the animals used in this study. The proportion of correct localization responses averaged over all twelve speaker positions is plotted over the course of twice-daily training with the earplug in place (Days 1–10). Symbols represent individual animals, control ferrets in black and lesion cases (NB ACh^−^) in magenta. Solid lines are the linear fits and shaded regions are the 95% confidence intervals for each group. Stimuli used were 1,000 ms broadband noise bursts (0.5–30 kHz) presented at five levels (56–84 dB SPL in 7 dB steps). Slopes = 6.4 (controls), 1.4 (NB ACh^–^); ANOVA (F_(__1__,___84___)_ = 20.153, *P* < 0.0001). Abbreviations: AEG, anterior ectosylvian gyrus; Cl, claustrum; D, dorsal; L, lateral; PEG, posterior ectosylvian gyrus; pss, posterior suprasylvian sulcus; sss, suprasylvian sulcus; V, ventral.

We explored the impact of cholinergic modulation on the auditory cortex by comparing functional properties of cortical neurons across both groups. Specifically, we analysed i) responses evoked by pure tones (frequency response areas) ii) spontaneous activity (level, regularity, synchronicity, bursting activity), iii) responses evoked by spatialized broadband noise presented in virtual acoustic space (VAS), both with normal inputs (open ears) and with a unilateral virtual earplug.

### 2.2 ME20.4-SAP injections in the ACh^−^ group

Bilateral injections in the NB of the immunotoxin ME20.4-SAP were performed in the NB ACh^–^ group (Fig. 1A). The immunotoxin comprises a monoclonal antibody - ME20.4 - against the p75 neurotrophin receptor (p75^NTR^) and saporin, which inhibits ribosome function when internalized in the p75^NTR^ cells (Pizzo et al., 1999). In adults, p75^NTR^ is expressed mainly in cholinergic cells from the basal forebrain (Kordower et al., 1989; Tremere et al., 2000; Leach et al., 2013; Bajo et al., 2014) (Fig. 1B,C).

Anesthesia was induced with medetomidine hydrochloride (0.022 mg/kg, i.m.; Domitor, Orion Pharma) and ketamine hydrochloride (5 mg/kg, i.m.; Ketaset, Fort Dodge Animal Health) and maintained with isoflurane (0.5–2.5%; IsoFlo, Abbott Laboratories, in 1–1.5 L/minute oxygen) delivered through a closed-loop ventilation system (Model 683; Harvard apparatus) after tracheal intubation. The radial vein was cannulated, and 5% glucose enriched saline solution was infused throughout the surgery at 5.0 ml/h. Heart rate, ECG, respiratory rate, O_2_ blood saturation, and end-tidal CO_2_ were continuously monitored. Body temperature was also monitored and kept at 38–39 °C using an electrical blanket with feedback from a rectal probe and a forced air warming blanket system (Bair Hugger, 3M).

The animal was mounted in a stereotaxic frame and after infusing the surgical field with local anesthetic (bupivacaine hydrochloride 0.25%; Marcain, Aspen), the skin was opened along the midline, the temporal muscles disinserted and retracted, and two small craniotomies were made that were centered at 19 mm anterior from the caudal end of the skull and 6 mm to the right and left from the midline (Bajo et al., 2014). The *dura mater* was opened and a glass micropipette (15–20 µm tip diameter), attached to a microinjector (Nanojet II; Drummond Scientific) and loaded with the immunotoxin, was lowered 8.11 mm ventral from the pial surface. The immunotoxin (27.6 nL containing 35.2 ng of immunotoxin) was injected at two different depths (200 µm apart) across two tracks separated 200 µm laterally in each hemisphere.

Intraoperative medication included atipamezole (0.5 mg/kg s.c.; Antisedan, Pfizer) to reverse medetomidine sedation once the animal was stabilized under isoflurane, methylprednisolone (20 mg/kg, s.c.; Solu-Medrol, Pfizer Inc.), buprenorphine (0.03 mg/kg, s.c.; Vetergesic, Alstoe Animal Health), meloxicam (0.2 mg/kg, s.c.; Metacam, Labiana Life Sciences), cimetidine (10 mg/kg, i.v.; Tagamet, GlaxoSmithKline), and atropine sulfate (0.06 mg/kg, s.c.; Atrocare, AnimalCare). Viscotears gel (Novartis) was used to protect the eyes from desiccation during surgery. Postoperative medication included buprenorphine (0.03 mg/kg, s.c.), meloxicam (0.05 ml, orally) and amoxycillin and clavulanic acid (20 mg/kg s.c.; Synulox, Zoetis).

After immunotoxin injections, the pieces of the cranium were replaced and stabilized with dental cement, the temporal muscles were repositioned by stitching them together at the midline, and the skin was sutured.

### 2.3 Virtual acoustic space

VAS stimuli were individually generated for each animal ahead of the recording experiments. Acoustic measurements were conducted in an anechoic chamber. After anesthesia was induced with medetomidine hydrochloride and ketamine hydrochloride, the radial vein was cannulated, and anesthesia was maintained with i.v. infusion of medetomidine hydrochloride and ketamine hydrochloride (0.022 mg/kg/h and 5 mg/kg/h, respectively) in 0.9% saline solution supplemented with 5% glucose.

Probe tubes were inserted at the entrance of the ear canals and connected to condenser microphones (E-4–211–2 microphone capsules; Sennheiser) so that the head-related transfer functions (HRTFs) could be measured for 12 azimuthal angles, corresponding to the arrangement of loudspeakers in the behavioral chamber in which the NB ACh^–^ animals were tested. To achieve this, clicks and broadband signals of 200 ms duration were presented from a loudspeaker (KEF T27, Falcon Acoustics) mounted on an articulated arm (65 cm radius, Tucker‐Davis Technologies) and positioned at 30° intervals in azimuth at 0° elevation. These acoustical recordings were used to generate VAS stimuli simulating both normal hearing conditions and a virtual earplug in the left ear, which was achieved by delaying and attenuating the input at that ear (Keating et al., 2013).

### 2.4 Neural recordings and auditory stimulation

Following the acoustic recordings, the animal was placed in a stereotactic frame. A metal bar was cemented onto the skull to free the ears and so that sounds could be delivered through earphones during the recording session. Bilateral craniotomies were performed, and the *dura mater* was removed to expose the auditory cortex bilaterally in the ectosylvian gyrus.

As previously described, depth of anesthesia, body temperature, heart rate, O_2_ blood saturation, and end-tidal CO_2_ were monitored and maintained in the physiological range throughout the recording duration. Atropine sulfate (0.06 mg/kg, s.c.) was administered at the beginning of the surgery to reduce the salivary secretions to facilitate intubation and to prevent bradycardia. Given the relative short half-life of atropine (about 2 h), by the time the neural recordings started, most of it would have been excreted because our ferret surgeries took 2–3 h. Dexamethasone (0.5 mg/kg, s.c.; Dexadreson; Intervet) to prevent brain edema was administered at the beginning of the surgery, and if required doxapram hydrochloride (4 mg/kg, s.c.; Dopram-V, Pfizer) to maintain respiratory rate.

Cortical activity was recorded using single shank Michigan probes (Neuronexus, Ann Arbor, MI, USA) with 32 recording sites linearly spaced at 50 μm intervals. All penetrations were oriented perpendicular to the pial surface and recordings from different parts of A1 were made by changing the position of the electrodes within the middle ectosylvian gyrus (Bizley et al., 2005).

Spontaneous and driven neural activity was recorded in respond to broadband noise bursts (BBN, 0.2–30 kHz bandpass and cosine ramped with a 10 ms rise/fall time, 100 and 400 ms duration, 10–90 dB sound pressure level, SPL, in 10 dB increments), which was generated afresh on each presentation, pure tones (100 ms duration, 0.5–30 kHz in 1/3rd octave steps, 10 to 90 dB SPL in steps of 10 dB, pseudorandomly presented with at least 3 presentations per frequency and level combination). The spatial sensitivity of cortical neurons was measured using VAS stimuli presented at 3 levels (56, 63 and 70 dB SPL; 30 repeats at each of the 12 azimuth locations pseudorandomly presented). Stimuli for the normal hearing condition and the simulated unilateral left earplug (virtual ear plug) were presented in separate blocks. Stimuli were generated with Tucker‐Davis Technologies System 3 hardware and Matlab (The Mathworks Inc.) and delivered through a pair of Panasonic earphone drivers (RP-HV298) mounted on plastic otoscope specula inserted into each ear canal. Calibrations of the sound-delivery system were performed ahead of the recording sessions using an 1/2th and an 1/8th inch condenser microphone (Brüel and Kjær, Nærum, Denmark).

### 2.5 Data analysis

Neural signals sampled at 25 kHz and bandpass filtered between 300 and 3,000 Hz were used to extract neuronal activity. Off-line spike sorting was performed in two steps. First, we used KlustaKwik2 (Kadir et al., 2014) for the unsupervised classification of potential clusters that were subsequently supervised using spikemonger (Rabinowitz et al., 2011), an in-house software package for Matlab. Candidate spikes were identified as voltage-threshold crossing events. An automated expectation-maximization algorithm sorted spikes by shape across up to five channels. Clusters that presented a clear and uniform spike shape and a marked reduction of events for times ≤1 ms in the autocorrelation histogram, indicative of a refractory period, were chosen for further analysis. If the reduction of events was close to zero, they were further classified as single units, otherwise they were classified as multi-units. More importantly, we found no difference between the responses of putative single units and small multi-unit clusters. Hereafter, unless specified otherwise, units refer to all clusters regardless of whether they were classified as single or multi-units. Only units that displayed acoustically-evoked activity to either broadband noise or pure tones were included in the analysis.

Data analysis was performed using custom Matlab scripts. Putative units were analysed only if spike counts in the first 50 ms following broadband noise onset were significantly different from the spike counts in a window of the same duration before stimulus onset (spontaneous firing rate) (paired *t*-tests, *P* < 0.05). Peri-stimulus time histograms (PSTHs) for each unit were calculated by summing action potentials, binned at 1 ms resolution, across all stimulus presentations. Peak response latencies were calculated as the time from stimulus onset to the bin with the maximum spike rate. Minimum response latencies were calculated as the time taken for the spike rate to first exceed 40% of the difference between peak and spontaneous firing rates.

Frequency response areas (FRAs) for responsive units were calculated as the sum of activity for each combination of level and frequency during pure tone presentations. Tuning curves were calculated using thresholds as determined by spontaneous firing rate + 0.2 × (peak firing rate) from which the characteristic frequency (CF, the frequency at which a response was observed at the lowest intensity level) and bandwidth, Q10 and Q30 (CF divided by the bandwidth at 10 or 30 dB above threshold) were calculated. Where more than one frequency was effective at the threshold, the CF was calculated as the logarithmically weighted mean.

Spatial tuning was determined for units that showed a statistically significant increase (*t*-test, *P* < 0.05) in firing rate in the onset response window (10–40 ms) compared to spontaneous activity in a window of the same duration. Rate-azimuth functions (RAFs) represented the response (spike count) for each unit as a function of azimuth and were used to calculate the preferred spatial direction and width of the spatial response profile. The overall preferred direction was estimated by calculating the centroid vector, which is the center of mass of the responses for each azimuthal location. The width of the spatial tuning was inferred from the equivalent rectangular receptive field (ERRF) (Lee and Middlebrooks, 2013). The ERRF was computed by integrating the area under the rate-azimuth function (RAF) and reshaping it as a rectangle whose height was the maximum azimuth response and equivalent area of the RAF (Lee and Middlebrooks, 2013). The modulation index estimates the depth of the firing rate modulation across spatial locations and was defined as

$$100\left(R_{max}-R_{min}\right)/R_{max}$$

where R_max_ and R_min_ are the maximum and minimum firing rate evoked for a particular location.

Data were checked for normality using the Kolmogorov–Smirnov test and parametric or non-parametric statistics test were used accordingly. Non-parametric statistical tests were used when data samples did not meet the normality criteria; Wilcoxon signed-rank test for paired comparisons and Wilcoxon rank-sum test for independent samples comparisons. Statistical significance was defined as *P* < 0.05. When data are presented in box plots, on each box, the central mark indicates the median, and the bottom and top edges of the box indicate the 25th and 75th percentiles, respectively. The whiskers extend to the most extreme data points not considered to be outliers, and the outliers are plotted individually using the '+' marker symbol.

### 2.6 Histological analysis

At the end of the recording experiment, animals were sedated with Domitor (0.022 mg/kg, i.m.), overdosed with pentobarbital sodium (400 mg, i.p., Euthatal, Merial Animal Health), and perfused transcardially with 0.9% saline and 4% fresh formaldehyde in 0.1 phosphate buffer, pH 7.4. The brains were removed, embedded in sucrose and cut on a freezing microtome into 45 µm thick coronal sections.

Histological serial sections across the auditory cortex were stained and used to confirm electrode locations. In NB ACh^–^ animals, sections were collected into 5 sets of serial sections; one set was stained for Nissl substance with 0.5% cresyl violet and used to confirm electrode locations. The other sets were used to visualize putative cortical cholinergic fibers visualized with acetylcholinesterase (AChE) histochemistry (Fig. 1A-C), and to immunostain against choline acetyltransferase (anti-ChAT) (Fig. 1A-C), p75^NRT^ (anti- p75^NRT^) (Fig. 1B, C) and neuron-specific nuclear protein (anti-NeuN).

AChE histochemistry was performed following Kamke et al. (2005a) and reported for ferret brain sections in Bajo et al. (2014). Briefly, after initial incubation in hydrogen peroxidase (0.1% H_2_O_2_) in 0.1 phosphate buffer saline (PBS) (pH=7.4), and washes in 0.1 M acetate/acetic acid buffer (pH=6.0), sections were preincubated in 10^−5^ M tetraisopropyl pyrophosphoramide (120 min at 37 °C), and then incubated for 30 min in 250 µM sodium citrate, 150 µM copper sulfate, 25 µM potassium ferrocyanide and 125 µM acetylthiocholine iodide. Finally, sections were reacted in 0.04% 3, 3′-diaminobenzidine tetrahydrochloride (DAB) and 0.3% ammonium nickel sulfate in Tris buffer with 0.003% H_2_O_2_.

For immunostaining, we used the following antibodies against choline acetyltransferase (ChAT, dilution 1:1000; rabbit polyclonal #AB143. Millipore Corporation), p75^NTR^ (dilution 1:500; mouse monoclonal AB-N07, Advanced Targeting Systems), and neural nuclei protein antibodies (NeuN; dilution 1:1000; mouse monoclonal anti- NeuN #Map377, Millipore Corp). Sections were incubated in 5% normal horse serum PBS for 1 h to reduce non-specific binding of the antibody. Primary antibody solutions were made with 0.1% Triton X_100_ PBS for ChAT and NeuN, and only PBS for p75^NTR^. Sections were kept in the primary antibody solutions for 72 h at +5 °C, followed by 90 min incubation in an ABC system (avidin-biotin complex, Vectastain Elite ABC; Vector Laboratories), and finally reacted using DAB and H_2_O_2_ (Leach et al., 2013; Bajo et al., 2014).

## 3 Results

We recorded spontaneous and driven activity in A1 in seven control ferrets and three ferrets in which cholinergic NB neurons were lesioned (NB ACh^–^). Sound-driven activity was recorded in response to pure tones of different frequencies and levels covering the ferret hearing range and to spatialized broadband noise presented in VAS to simulate the 12 azimuthal locations used in the sound localization task in which the NB ACh^–^ ferrets were tested.

For eithical reasons, control animals were not subjected to a sham surgery to mimic the experience of the NB ACh^–^ animals since previous studies in our laboratory have shown that animals in which cranial surgeries have been carried out to inject substances in different parts of the brain (Bajo et al., 2010, 2019; Leach et al., 2013) or to place drug-free, slow-release polymers over the auditory cortex (Smith et al., 2004; Nodal et al., 2012) did not show any change in their sound localization or spatial learning abilities, suggesting that surgical manipulations per se do not affect the response properties of the cortical neurons.

1.1 Injections of the ME20.4-SAP immunotoxin in the nucleus basalis bilaterally produce cholinergic cell loss that correlates with behavioral impairment

The recordings were made approximately 6 months following the cholinergic lesions in order to allow time for the behavioral study to be carried out. Our behavioral data showed no indication of any recovery of function during this time and previous studies using injections of the same immunotoxin into the basal forebrain found no changes in AChE staining in the neocortex and hippocampus over a comparable period (Leanza et al., 1995). This does not rule out the possibility, however, that compensatory changes in cholinergic function may have taken place in other brain areas (Change and Gold, 2004). Perfusion and brain histology followed the final recordings.

To assess cholinergic cell loss, we quantified the number of cholinergic cells in the NB bilaterally in the NB ACh^–^ and control cases (Fig. 1B, C). The proportion of p75^NRT^ cells lost in the NB was at least 84%. As result of cholinergic cell loss in the NB, AChE staining in the auditory cortex was weaker in NB ACh^–^ ferrets than in controls (Fig. 1B, C; for stereological quantification see Table 1 in Leach et al., 2013), even though the ACh staining does not exclusively label cholinergic axons.

The ferrets with cholinergic lesions exhibited a duration-dependent change in their ability to localize broadband noise bursts in the horizontal plane, with significantly lower scores achieved by the NB ACh^–^ group than control ferrets for shorter stimuli (40–500 ms, F_(1,19)_ = 21.67, *p* < 0.001) but not for longer stimuli (1000–2000 ms, F_(1,19)_ = 3.81, *p* = 0.066) (Fig. 1D) (see Leach et al. (2013) for a full analysis of these data). The ability of the NB ACh^–^ ferrets to adapt their localization responses to the unilateral conductive hearing loss produced by plugging one ear was also compromised (Fig. 1E). Adaptation slopes were significantly different from 0 (slopes: controls, 6.4, NB ACh^–^, 1.4) and significantly different from each other (ANOVA; F_(__1, 84)_ = 20.153, *P* < 0.0001).

The selectivity of the immunotoxin for cholinergic neurons and the specificity of NB immunotoxin injections in producing these behavioral deficits in the NB ACh^–^ cases were confirmed in Leach et al. (2013). Briefly, two ferrets received bilateral injections of saline solution in the NB and in one ferret the ME20.4-SAP immunotoxin was injected into the caudate nucleus; in neither case was any behavioral impairment or cholinergic cell loss observed (see Leach et al., 2013 for more details). In addition, ME20.4-SAP immunotoxin injected in the NB produced specific cholinergic cell loss in the NB but not in other cholinergic groups in the basal forebrain as the medial septum (Leach et al., 2013).

1.2 Frequency tuning of auditory cortical neurons is not affected by cholinergic cell loss in the nucleus basalis

**Fig. 2:**
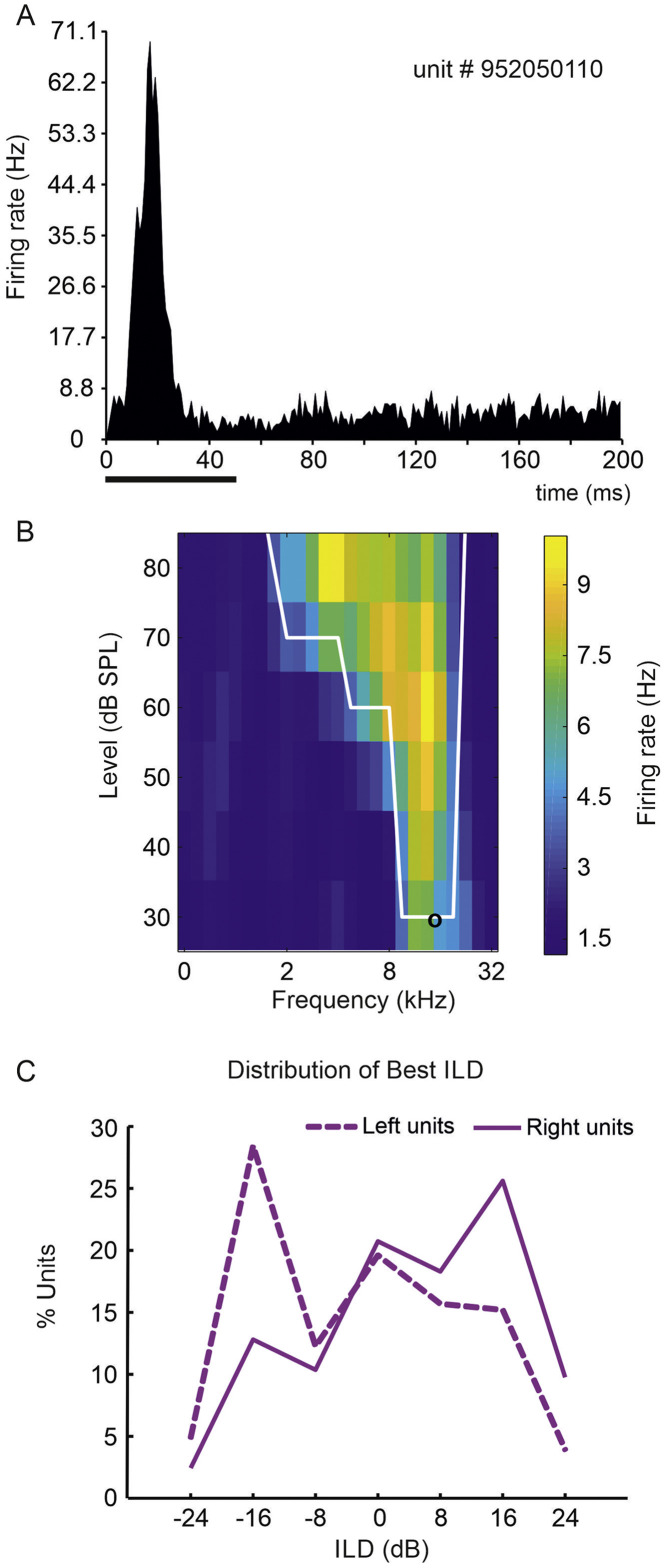
Frequency tuning and interaural level difference sensitivity in the NB ACh^−^group.

A1 units recorded in the control (*n* = 264) and NB ACh^–^ (*n* = 360) ferrets were tuned for sound frequency and showed a contralateral preference (Fig. 2), in agreement with previous descriptions in the ferret (Bizley et al., 2005; Keating et al., 2015). The responses of these units were classified as onset, offset or onset-offset types, depending on whether the evoked activity in the onset and/or offset response windows was significantly higher than the spontaneous activity. The majority of units were classified as onset-offset (controls 88.6%, NB ACh^–^ 77.3%), with the offset response being much smaller than the onset response. Units responding exclusively to the offset of the stimulus were the least numerous (control 2.2%, NB ACh^–^ 3.9%). Onset peak response latencies for control units (median 20 ms) were marginally longer (NB ACh^–^ median 19 s) (Wilcoxon rank-sum test *Z* = 2.252; *p* = 0.0243). No difference in offset peak latencies were found between the groups (controls: median 78 ms; NB ACh^–^: 79 ms) (Wilcoxon rank-sum test *Z* = −0.156; *P* = 0.8761; stimulus duration 50 ms). The Neuronexus probes we used have 32 recording sites linearly spaced at 50 mm intervals that were always placed approximately perpendicular to the cortical layers, leaving the most superficial recording site close to pial surface. The activity of neurons across all layers of the cortex should therefore have been sampled, although since relatively few units were recorded in the superficial layers, it is not possible to say whether there were differences in the laminar distribution of the responses.

**A**, Peri-stimulus time histogram of a cortical neuron (unit # 952050110) recorded from the left A1 of a NB ACh^–^ animal (0952) in response to the pseudorandom presentation of 50-ms pure tones of different frequencies and levels (30 repeats). The stimulus presentation is indicated by the black horizontal line. **B**, Frequency response area (FRA) for the same unit in A based on the number of spikes counted in the onset response window (10–40 ms). The firing rate is indicated by the color scale, the white line indicates the limits of the FRA and the circle is the unit's characteristic frequency. **C**, Distribution of best ILDs showing for neurons in the NB ACh^–^ group. There were no differences between hemispheres (left *n* = 204, right *n* = 164; Chi-Squared test χ^2^(6,1)= 0.0462, *P* = 1) and best ILDs were mainly contralateral. Negative ILD values (left-right) indicate greater intensity at the right ear.

**Fig. 3:**
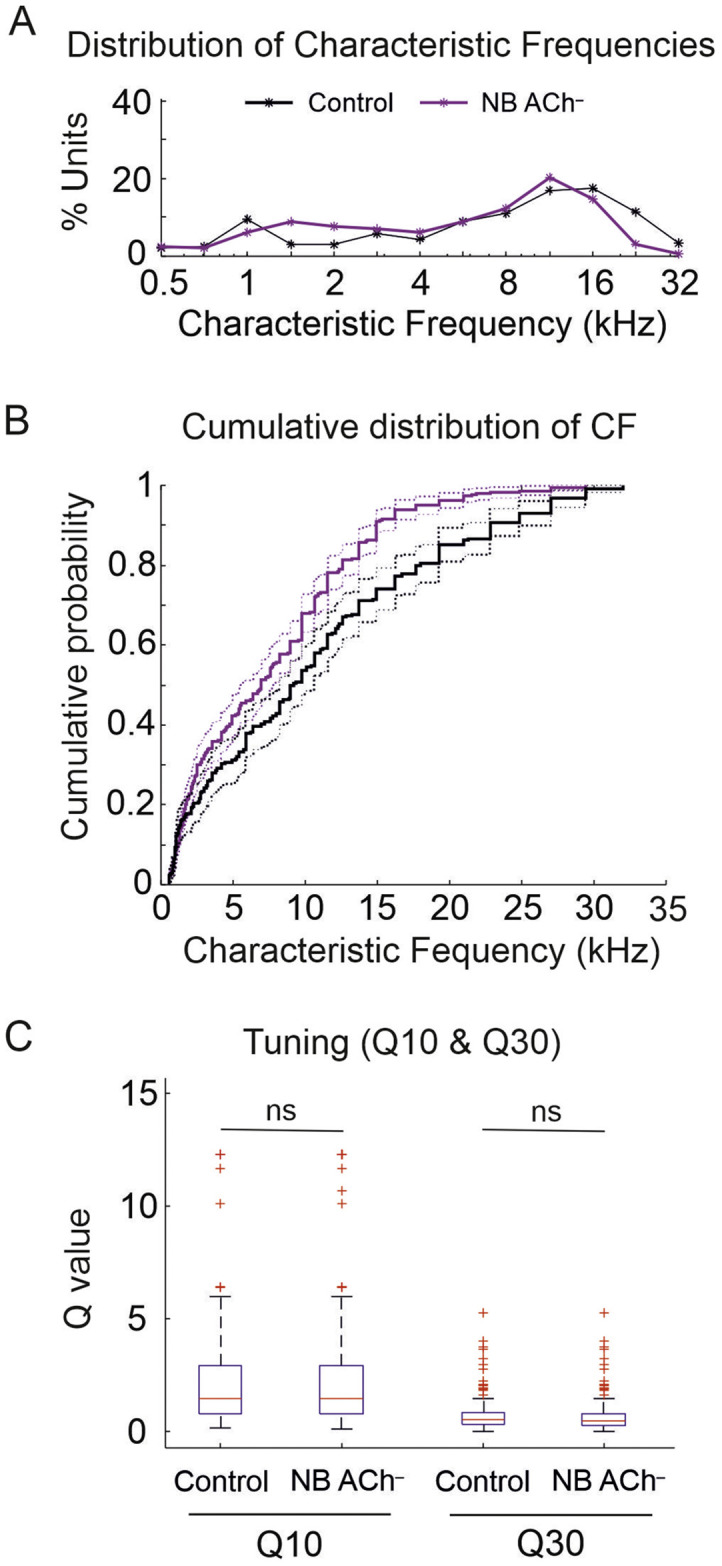
Spectral tuning properties.

To determine whether the depletion of ACh affected the frequency tuning properties of cortical neurons, we compared the tuning properties of units recorded from the NB ACh^–^ group with those from the control group. However, to ensure that differences in frequency tuning properties were not biased by differences in recording locations within the middle ectosylvian gyrus across animals, we first compared the distributions of characteristic frequencies (CF) in half-octave intervals in each animal group (Fig. 3A). The distribution of CFs in the two groups was similar (Fig. 3A) (controls: median CF 9.2 kHz; NB ACh^–^: median CF 6.9 kHz) and the cumulative distributions of CF were not significantly different between the two groups (Chi-squared test, χ^2^= 0.0498; *P* = 1) (Fig. 3B).

**A**, Distribution of recorded units according to their characteristic frequency (CF) in half-octave-wide bins for both groups (controls *n* = 264; NB ACh^–^*n* = 360). **B**, Cumulative distributions of unit CFs were not statistically significant different between the control and NB ACh^–^ groups. **C**, Frequency tuning expressed as Q values for 10 and 30 dB above the threshold at the CF.

No differences were found between the controls and the NB ACh^–^ ferrets in the thresholds of A1 units estimated at their CFs (median for both groups = 30 dB SPL, Wilcoxon rank-sum test *Z* = 0.0381; *P* = 0.9696) or in the width of their frequency tuning, estimated by the Q10 (Wilcoxon rank-sum test *Z* = 0.7619; *P* = 0.446) and Q30 (Wilcoxon rank sum test *Z* = 1.1177; *P* = 0.2637) (Q10 and Q30 = CF/bandwidth 10 and 30 dB above the threshold at the CF, respectively) (Fig. 3C).

1.3 Cholinergic deafferentation of the auditory cortex does not affect neural spontaneous activity or synchrony

**Fig. 4:**
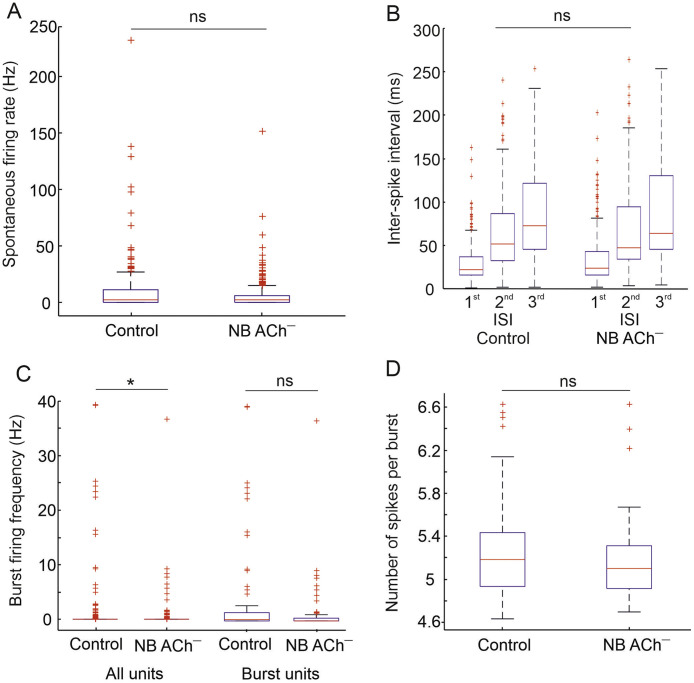
Spontaneous activity and firing patterns in auditory cortical neurons after cholinergic deafferentation.

In addition to the tone-evoked activity, we also explored whether ACh depletion affected the spontaneous activity of cortical neurons. No difference was found in the median spontaneous activity between NB ACh^–^ units (*n* = 327) and control (*n* = 402) units (controls: 2.81 Hz; NB ACh^–^: 2.63 Hz; Wilcoxon rank-sum test, *Z* = −1.56; *P* = 0.1178) (Fig. 4A). To further explore whether cholinergic deafferentation had an effect on spontaneous firing behavior beyond a simple change in the overall firing rate, we examined the firing pattern by calculating the first, second and third-order inter-spike intervals (ISI) for each unit and used a non-parametric method to detect spike bursts (following Gourévitch and Eggermont, 2007). Population median values of ISIs did not change as a result of ACh depletion (Fig. 4B) (1st, 2nd and 3rd ISI Wilcoxon rank test, *P* = 0.8961, *P* = 0.8137, *P* = 0.9752). However, we observed a statistically significant drop (Chi-squared test, χ^2^= 10.258; *P* = 0.0014) in the percentage of units that exhibited bursting behavior in the NB ACh^–^ group (control 28.01%; NB ACh^–^ 17.91%). In addition, units in the NB ACh^–^ group exhibited a lower burst firing frequency (controls: 0.91 Hz ± 4.42; NB ACh^–^ 0.247 Hz ± 2.04) (Wilcoxon rank-sum test, *Z* = −2.32; *P* = 0.0203) (Fig. 4C).

**A,** No differences were found in spontaneous activity between units from the cholinergic lesion group (NB ACh^–,^*n* = 402) and units in the control group (*n* = 327) (Wilcoxon rank-sum test, not significant, ns *p* = 0.1178). Box plot showing the median (red line), interquartile range (box), the range of the most extreme values not considered as outliers (whiskers), and outlier values (crosses). **B**, First, second and third order inter spike intervals were unaffected by cholinergic deafferentation. **C**, Burst firing frequency was analysed across all units (left) (Wilcoxon rank-sum test * *p* = 0.0203) and in units for which at least one burst was identified (right). **D**, Plot showing the number of spikes per burst. For both burst firing frequency (**C**) and the number of spikes per burst (**D**), no differences were found between units from the NB ACh^–^ and the naïve control groups, although the percentage of burst-type neurons decreased with acetylcholine deafferentation from 28.01% to 17.91% (χ^2^= 10.258; *p* = 0.0014).

To determine whether the lower burst frequency could be explained by the lower number of burst-firing neurons, we also compared the burst frequency in the neurons for which at least one burst was identified. Although the burst frequency was larger in the control group than in the NB ACh^–^ group (3.26 Hz ± 7.90 versus 1.38 Hz ± 4.68), these differences were not statistically significant (Wilcoxon rank-sum test, *Z* = −1.4035; *P* = 0.1605) (Fig. 4C). Therefore, it seems that deafferentation of ACh reduces the proportion of auditory cortical units that exhibit burst firing without affecting the characteristics of the bursting activity *per se*. The number of spikes per burst (median controls: 5.18; NB ACh^–^ 5.10) did not vary between groups (Wilcoxon rank-sum test, *Z* = −1.4035; *P* = 0.1605) (Fig. 4D), suggesting that ACh does not affect the intrinsic characteristics of burst firing when it occurs.

Neural synchrony was estimated by calculating the cross-correlation between the spike trains from pairs of units recorded simultaneously. Spike trains recorded for 400 ms in response to 200-ms broadband noise bursts were binned into 1 ms wide bins. The first 220 ms of each sweep were used to calculate the synchrony for driven activity and the last 180 ms for synchrony in the spontaneous activity. Cross-correlation histograms were produced using the xcorr function in Matlab according to the method described by Brosch and Schreiner (1999), with a maximum temporal shift of ±20 ms. From each cross-correlogram, the maximum value and its temporal shift were extracted and compared for spontaneous and driven activity within and between groups. For pairs of units from control animals, no differences were observed between the maximum correlation value (spon: 0.741± 0.120; driven: 0.743± 0.180). However, for NB ACh^–^ unit pairs, the maximum correlation values were statistically greater (Wilcoxon rank-sum, *Z* = 5.6474, *P* < 0.0001) for the driven period (0.815 ± 0.157) than the spontaneous period (0.7447 ± 0.1889). Comparing the two groups, NB ACh^−^units showed a significantly greater maximum normalized correlation value (0.780 ± 0.177) than controls (0.742 ± 0.211) (Wilcoxon rank-sum, *Z* = 5.2923, *P* < 0.0001). However, the temporal shift of the maximum correlation between pairs of units did not differ either between groups (Wilcoxon rank-sum, *Z* = −0.0029, *P* = 0.9977) or between driven and spontaneous periods within each group. It seems that cholinergic deafferentation led to more similar neural responses than those observed in control animals without altering the time course of the neural activity.

1.4 Spatial sensitivity of cortical neurons is not altered by loss of cholinergic input from the nucleus basalis

We examined whether cortical cholinergic depletion had an effect on the spatial sensitivity of A1 neurons by comparing the responses of units recorded from NB ACh^–^ ferrets with those from behaviorally naïve controls to sounds presented in virtual acoustic space simulating either normal binaural inputs or monaural earplugging. We chose to use behaviorally naïve controls since training ferrets with one ear occluded normally results in a compensatory improvement in sound localization accuracy that relies on A1 (Bajo et al. 2019), and, at least when altered spatial cues are experienced during development, is associated with compensatory changes in the cortical processing of monaural and binaural spatial cues (Keating et al., 2013, 2015). Any change in the spatial properties of units in NB ACh^–^ ferrets versus the control animals should therefore be due to the cortical depletion of acetylcholine in the former group, rather than effects of learning in the controls.

**Fig. 5:**
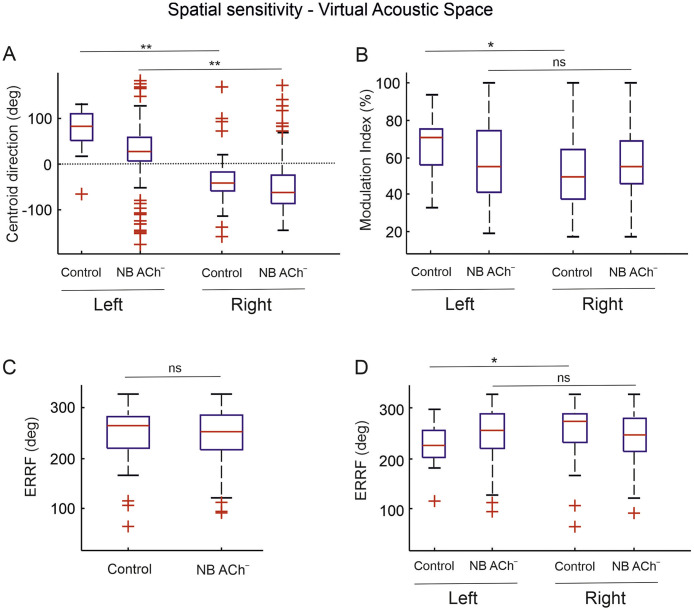
Spatial tuning properties to sound presented in the virtual space simulating normal hearing conditions.

Most of the units in both controls and NB ACh^–^ ferrets showed a preference for ILDs favoring the contralateral ear (Fig. 2C). This contralateral preference was equally apparent in both hemispheres and confirmed when spatialized noise bursts were presented using individualized VAS (controls: *n* = 60, NB ACh^–^^:^ *n* = 265) (Fig. 5). This contralateral preference is indicated by the direction of the median centroid vector (Fig. 5A). Units recorded from the left hemisphere had a positive (rightward from midline) centroid direction (median/ iqr; controls: 80.8°/ 59.2; NB ACh^–^: 26.1°/ 53), whereas for right hemisphere units, median centroid orientations were negative (leftwards to the midline) (controls: −43.1°/ 42 NB ACh^–^: −63.9°/ 63.3) (Fig. 5A).

**A**, Spatial centroid directions for controls (*n* = 60) and NB ACh^–^ (*n* = 265) groups divided according to whether units were recorded in left or right A1. The centroid direction values indicate a contralateral preference in both hemispheres and therefore differed between the left and right A1 units in both groups (Wilcoxon rank-sum test *p* < 0.001 **). **B**, Spatial modulation index, which quantifies the difference in activity between the spatial locations that evoked the largest and smallest responses. In the control group, left A1 units exhibited a greater modulation than the right A1 units (Wilcoxon rank-sum test *p* < 0.05 *) whereas for the NB ACh^–^ units there was no difference between left and right units. **C** and **D**, Equivalent rectangle receptive field (ERRF) for all units in each group (**C**) and within each group (**D**) divided according to the hemispheric recording location of the units. No differences between groups were found, but when broken down by hemisphere the right units in the control group showed broader spatial tuning (Wilcoxon rank-sum test *p* < 0.05 *).

Despite this overall contralateral preference, most of the units responded to all tested spatial locations. To assess the spatial tuning of the units, we measured their modulation index, which quantifies the difference between the activity at the locations where the largest and smallest responses were obtained (Fig. 5B) (median/ iqr; controls: 55.8%/ 32.2; NB ACh^–^: 54.9%/ 29.0), as well as the width of the rate-azimuth functions parametrized by the equivalent rectangular receptive field (ERRF). The ERRF values highlight the broad azimuth tuning in both groups (median/ iqr; controls: 264.2°/ 64.9; NB ACh^–^: 252.1°/ 68.4) (Fig. 5C,D). Neither the modulation index (Wilcoxon rank-sum, *Z*= −0.319, *P* = 0.749) nor the ERRF (Wilcoxon rank-sum *Z* = 0.6641, *P* = 0.506) differed between the control and NB ACh^–^ units. Considering all the parameters together, these results indicate that depletion of cholinergic inputs from the NB into the auditory cortex did not affect the spatial sensitivity of the cortical units or their contralateral preference.

**Fig. 6:**
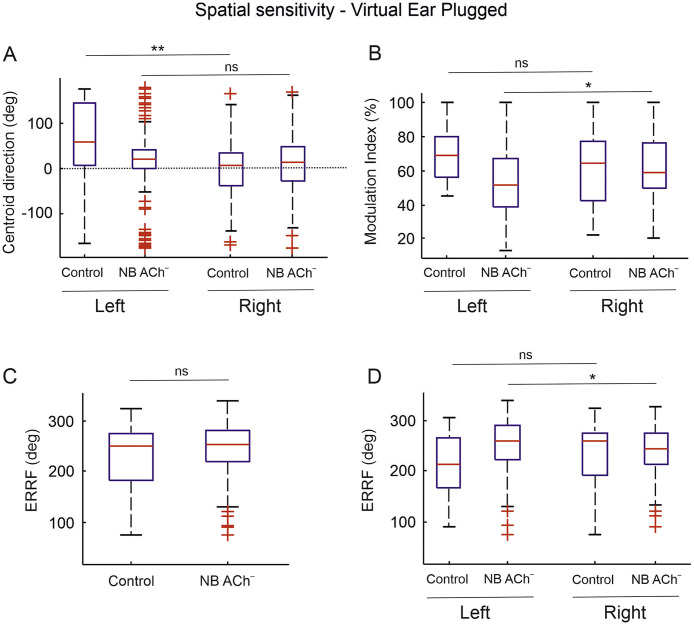
Spatial tuning properties in response to sound presented in virtual space simulating a left ear plugged condition.

**Fig. 7:**
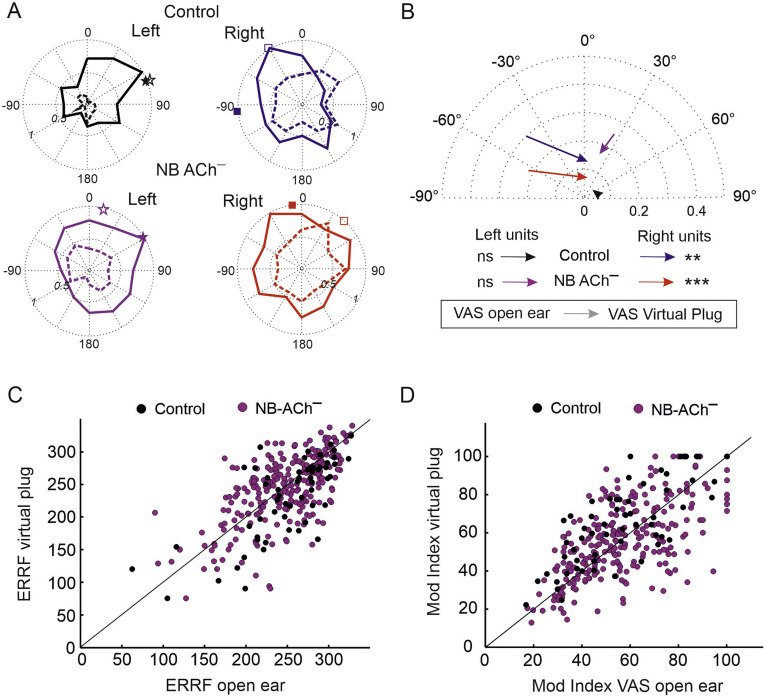
Changes of spatial properties introduced by a left virtual earplug.

Presentation of stimuli that simulated a unilateral conductive hearing loss in the left ear resulted in a dramatic change in the spatial tuning of the cortical units in both control and NB ACh^–^ groups (Fig. 6, Fig. 7). The virtual earplug changes the relationship between the binaural cue values and azimuthal locations. Behaviorally, plugging one ear (the left in this case) biases localization responses towards the side of the contralateral open right ear, with larger localization errors in the left, ipsilateral hemifield (Leach et al., 2013 and Sanchez Jimenez et al., 2023). Normally, ferrets adapt to the altered inputs with training, so these errors gradually disappear over the course of monaural occlusion (Leach et al., 2013 and Sanchez Jimenez et al., 2023; Fig.1E). Because the NB ACh^–^ showed greatly reduced adaptation, however, we predicted that the virtual earplug would have a very similar effect on the azimuth sensitivity of the A1 units in these animals and the untrained control group.

**A**, Spatial centroid directions in the presence of a virtual earplug in the left ear for controls (*n* = 60) and NB ACh^–^ (*n* = 265) groups divided by the cortical recording location. Units in the left cortex still exhibited a contralateral preference, as in normal hearing conditions (Fig. 5), whereas units in the right hemisphere lost their clear contralateral preference. Those changes were more pronounced in the NB ACh^–^ as the lack of a difference between the two hemispheres indicates. **B**, Spatial modulation index in the presence of a virtual earplug, which resulted in a significant difference between hemispheres in the cholinergic lesioned group (Wilcoxon rank-sum test *p* < 0.05 *) but not in the control group. **C** and **D**, ERRF values in the presence of a virtual earplug for all units in each group (**C**) and within each group (**D**) divided by the hemispheric location of units. No overall difference was found between the groups (**C**), but when broken down by hemisphere (**D**) left units in the NB ACh^–^ group showed slightly broader tuning (Wilcoxon rank-sum test *p* < 0.05 *).

**A,** Example units from the control group (top row, black and blue lines) and NB ACh^−^ (bottom row, purple and brown lines) group recorded from left and right auditory cortex (A1) showing their responses (firing rate normalized to the maximum response) to broadband noise presented under normal hearing conditions (solid lines) and in the presence of a virtual plug in the left ear (dashed lines). Note that the responses decrease with the virtual earplug and show shifts in spatial preference, as indicated by the direction vectors (symbols located on the periphery of each graph, filled symbols for normal hearing conditions and open symbols in the presence of a virtual earplug). **B**, changes in median centroids caused by the left virtual earplug (controls *n* = 60) and NB ACh^–^*n* = 265). The direction of the arrows indicates the direction of the change from the open ear condition to the left virtual plug condition. For both groups and hemispheres, the centroids become less lateralized. The right A1 units, normally tuned to the spatial positions ipsilateral to the earplug were more affected than the left A1 units (Wilcoxon signed-rank test, ** *p* < 0.001, *** *p* < 0.0001). **C**, ERRF for all the units for the open ear and virtual earplug conditions. **D**, Spatial modulation index (Mod Index) for all the units for the open ear and virtual earplug conditions.

The virtual earplug altered the spatial sensitivity of A1 units in both groups, with the largest changes observed, as expected, in the contralateral right hemisphere (Fig. 6, Fig. 7). The centroids of A1 units recorded in the right hemisphere shifted from contralateral to ipsilateral (Fig. 6A) (median/ iqr; controls: from −43.1°/ 42 to 6.9°/ 72.7; NB ACh^–^: from −63.9°/ 63.3 to 13.8°/ 77.3). Units in the left hemisphere showed smaller changes and maintained a contralateral preference (median/ iqr; controls: from 80.8°/ 59.2 to 56.5°/ 137.2; NB ACh^–^: from 26.1°/ 53 to 20.3°/ 42.2). The magnitude of the shift in centroid direction across groups depended on the degree of contralateral preference shown in normal hearing conditions (Fig. 7A). The ferrets with cholinergic lesions showed the same trend as the controls when the virtual earplug was present, suggesting that previous experience of wearing an earplug does not result in long-term changes in spatial response properties, at least if that experience was not behaviorally relevant, as indicated by the very limited adaptation in the localization responses of the NB ACh^–^ group (Fig. 1E).

The virtual earplug had little overall effect on the modulation index or ERRF values in either the control or NB ACh^–^ groups (Fig. 5, Fig. 6, Fig. 7). Small but significant hemispheric differences were found in the way the virtual earplug affected the spatial tuning parameters in the two groups (Fig. 6), which may either reflect sampling differences or the prior experience with an earplug of the ferrets with cholinergic lesions.

## 4 Discussion

In this study, we examined the response properties of neurons in the auditory cortex of ferrets in which much of the cholinergic input from the basal forebrain had been selectively eliminated. These animals exhibited deficits in the localization of brief sounds and in their ability to adapt with training to altered auditory spatial cues following occlusion of one ear (Fig. 1, Leach et al., 2013). The primary auditory cortex plays an essential role in this behavioral plasticity (Bajo et al., 2019), which involves both learning to place greater weight on the unchanged monaural spectral cues available at the open ear as well as compensatory changes in binaural cue sensitivity (Kacelnik et al., 2006; Keating et al., 2016; Sanchez Jimenez et al., 2023). This is supported by the finding that cortical neurons recorded in ferrets that adapted behaviorally to chronic occlusion of one ear during development can show reweighting of different spatial cues in the presence of unilateral conductive hearing loss and a systematic shift in their sensitivity to interaural level differences (Keating et al., 2013; 2015; 2016). The spectral and spatial response properties of the cortical neurons recorded in the animals with cholinergic lesions were very similar to those of naïve control ferrets, suggesting that ACh is not required for the maintenance of these properties, but does play a crucial role in mediating the plasticity that underpins the ability to relearn to localize sound accurately when spatial cues are disrupted by occlusion of one ear.

Numerous studies have shown that the cholinergic modulation of cortical neurons is involved in various cognitive processes that include arousal, attention, learning and memory induction (Hasselmo and Sarter, 2011; Weinberger, 2015). One of the best studied examples of the potential role of cholinergic inputs in learning and memory is provided by the finding that repeated pairing of sounds with NB stimulation can induce stimulus-specific changes in neuronal responses and representational plasticity at both cortical (Bakin and Weinberger, 1996; Kilgard and Merzenich, 1998a, b; Bao et al., 2003; Froemke et al., 2007) and subcortical levels of the auditory system (Ma and Suga, 2003; Zhang and Yan, 2008). The modulatory influence of cholinergic basal forebrain inputs on cortical processing has been shown to be associated with improvements in sound detection and discrimination (Froemke et al., 2013), further implicating ACh in auditory perceptual learning.

Cholinergic actions on auditory cortical neurons are mediated by both muscarinic and nicotinic receptors (Metherate, 2011) and may influence thalamocortical transmission as well as the activity of both excitatory and inhibitory cortical cell types (Metherate, 2011; Nelson and Mooney, 2016). There are therefore several potential ways in which ACh release can influence sound-evoked responses in the auditory cortex. Indeed, a range of effects on neuronal thresholds and frequency selectivity have been described following application of cholinergic agonists (McKenna et al., 1989; Metherate, 2011) or stimulation of cholinergic axons from the basal forebrain (Nelson and Mooney, 2016). Furthermore, it has recently been reported that cholinergic basal forebrain neurons transmit rapid, behaviorally-neutral auditory signals spanning a range of frequency preferences to cortical neurons (Zhu et al., 2023). These phasic cholinergic inputs undergo tonic changes related to behavioral and brain states and could potentially provide the basis for triggering stimulus-specific plasticity or attentional effects on the tuning of auditory cortical neurons.

Given the extensive evidence for a critical involvement of cholinergic modulation of cortical activity in various aspects of cognition, including the role of the auditory cortex in learning and memory encoding, it is essential to investigate how ACh influences neural processing and plasticity when the stimuli are behaviorally relevant. However, it is also important to determine how the response properties of auditory cortical neurons are affected by a loss of basal forebrain cholinergic input, particularly since cholinergic cell loss is associated with aging (Altavista et al., 1990) and dementias such as Alzheimer's disease (Hasselmo and Sarter, 2011; Hampel et al., 2018). We chose to compare the various physiological properties of auditory cortical neurons recorded in ferrets with cholinergic lesions with those measured in naïve, untrained animals rather than to use ferrets with intact cholinergic inputs that had successfully adapted with training to unilateral conductive hearing loss. This is because the process of adaptation would be expected to alter the spatial response properties of cortical neurons, as shown by monaural occlusion during development (Keating et al., 2013; 2015; 2016), from which it would be difficult to conclude whether any differences are due to the impaired adaptation in the ferrets with ME20.4-SAP immunotoxin lesions of the NB, as opposed to a direct effect of loss of cholinergic inputs on those properties.

Given the association between cholinergic neurons and arousal, reward, motor function, attention and other cognitive functions, we wanted to rule out the possibility that any changes in auditory cortical processing measured in ferrets with cholinergic lesions were indirect consequences of differences in brain or behavioral state between these animals and the control group. We therefore opted to carry out the recordings under general anesthesia, as was the case in the majority of studies in which the modulatory effects of pairing sounds with stimulation of the basal forebrain have been measured (e.g. Froemke et al., 2007; Reed et al., 2011). We used a cocktail of medetomidine and ketamine as anesthetic agent. Both drugs have a strong effect on arousal (Brown et al., 2011). Medetomidine induces a state similar to non-REM sleep by acting on α2 adrenergic receptors mainly in the locus coeruleus (LC). The hyperpolarization of LC cells may reduce excitatory inputs to the basal forebrain and disinhibit the hypothalamic preoptic area, which then inhibits the ascending arousal centres (Nelson et al., 2002, 2003). Ketamine alters arousal by binding preferentially to NMDA receptors on GABAergic inhibitory interneurons (Olney et al., 1999; Seamans, 2008), creating a dissociative state with aberrant cortical activity that lacks normal spatial and temporal coordination. It is therefore possible that this commonly used anesthetic regime might have contributed to the lack of differences in the basic properties of cortical units recorded in cases with or without cholinergic input. However, very similar facilitatory effects of basal forebrain stimulation on tone-evoked responses in the auditory cortex have been found in awake rats to those reported under anesthesia (Hars et al., 1993), so we would not expect this to be a major factor affecting our results.

In vitro work has shown that 70–80% of cells in the basal forebrain are mainly silent at rest (Hedrick and Waters, 2010). Our results indicate that under medetomidine/ketamine anesthesia, spontaneous activity of cortical neurons is low and not particularly affected by loss of cholinergic inputs from the nucleus basalis, other than a higher proportion of bursting units in the control group than in the NB ACh^-^ group. There is growing evidence that spontaneous activity and aspects of stimulus coding in the auditory cortex can be affected by general anesthesia (Zurita et al., 1994; Banks et al., 2018; Filipchuk et al., 2022; Middlebrooks et al., 2023), but since both groups of animals were anesthetized in the same way during the recordings, the key difference would have been the loss of most of the cholinergic innervation of the cortex in the NB ACh- group.

When comparing different groups of animals, it is always desirable that the only difference between them would be the experimental manipulation, in this case, the ablation of cholinergic input from the NB, to discount other factors that might contribute to the differences observed. However, that is not always possible; in this case, the animals in the control group had not experienced a previous cranial surgery whereas the NB ACh^–^ group had undergone a previous surgery so that bilateral immunotoxin injections could be made to target cholinergic cells in the NB. We did not perform a sham surgery in the control animals since in previous studies we have not observed any changes in the sound localization and learning abilities of animals that underwent cranial surgeries with different levels of invasiveness involving the auditory pathway (Nodal et al., 2012; Bajo et al. 2019) or other structures (Leach et al. 2013); therefore we considered it reasonabe to assume that previous surgical intervention is not a contributory factor. It is also important to note that all animals in both groups were administered at the beginning of the surgery with atropine, an antimuscarinic drug that can cross the blood brain barrier, to reduce salivary secretions for intubation and anesthesia-related bradycardia; because of its relative short half-life (2 h), however, most of it would have been excreted by the time the neural recordings started.

We found that the temporal firing patterns of auditory cortical neurons were similar in the control group and in the ferrets with cholinergic lesions. While significantly fewer burst type neurons were observed following ACh depletion, and greater synchrony was found between pairs of recorded units, no differences were found in response thresholds or frequency tuning. The distribution of CFs determined from the frequency response areas was indistinguishable between the control and lesion groups. A lack of effect of cholinergic lesions on the tonotopic organization of A1 has also been reported in cats in which a partial lesion of the cochlea was made in one ear (Kamke et al., 2005b). In this study, CFs varied systematically in the normal fashion in response to stimulation of the unlesioned ipsilateral ear, whereas the cortex contralateral to the lesioned ear had an expanded representation of the lesion-edge frequencies, which closely resembled the frequency reorganization induced by unilateral cochlear lesions in animals with intact cholinergic inputs. Our data differ in that behavioral adaptation to conductive hearing loss in one ear is dependent on basal forebrain cholinergic modulation, but, in both studies, spectral response properties were unaffected by the loss of cholinergic input alone.

Of more relevance to the behavioral deficits observed in the ferrets with cholinergic lesions is the effect on the spatial response properties of auditory cortical neurons. A symmetrical distribution of best ILDs was found in each hemisphere, each showing a clear preference for the contralateral hemifield. This is what is found in the auditory cortex of control ferrets and differs from the compensatory shift in ILD sensitivity that occurs in ferrets that adapt to long-term monaural occlusion during development (Keating et al., 2015). We measured azimuth sensitivity using broadband noise presented in virtual acoustic space that was individualized using acoustical measurements of the HRTF in each ferret, and found no differences in either the centroid direction vectors or bandwidth between the ferrets with cholinergic lesions and the naïve control group. Together, these findings suggest that the cortical spatial responses measured under normal hearing conditions in the ferrets with cholinergic lesions are normal. This is consistent with their limited adaptation to unilateral conductive hearing loss. Nevertheless, it is possible that the small differences observed in spatial response parameters between the groups when the hearing loss was simulated with a virtual earplug might be related to the very modest improvement in localization accuracy that took place with training over the course of the period of monaural occlusion in the ferrets with cholinergic lesions (Leach et al., 2013).

These data do not shed light on the basis for the reduced ability of the ferrets with cholinergic lesions to localize very brief sounds compared to normal controls. It is possible that rather than a specific change in spatial processing, this actually reflects a deficit in spatial attention or in remembering the location of the loudspeaker from which the stimulus had been presented (Leach et al., 2013). Although we focused on the effects of cholinergic input to the auditory cortex, cholinergic neurons in the NB project to the entire neocortex and the amygdala (Thiele 2013). They therefore potentially influence a wide range of perceptual and cognitive processes (Qi et al., 2021), which may contribute to the learning deficits described in awake ferrets (Leach et al. 2013). Because the recordings were performed in A1 under anesthesia, it is unlikely that the effects observed are due to changes in the cholinergic inputs to other cortical areas. To obtain a clearer picture of the role of the basal forebrain during active listening, it will be necessary to record the activity of auditory cortical neurons in awake, behaving animals and to combine this with simultaneous manipulations of their cholinergic input.

How cholinergic signals mediate cortical (or subcortical) plasticity to enable animals with a unilateral earplug to relearn to localize sounds accurately is unclear. However, in vitro recording experiments have shown that ACh release can induce persistent firing in layer 5B auditory cortical neurons that project to the inferior colliculus, but not in layer 5B neurons that project to the contralateral cortex (Joshi et al., 2016). Since the integrity of the corticocollicular projection is essential for adaptation to unilateral conductive hearing loss (Bajo et al., 2010), this finding identifies a potential circuit by which this neuromodulator might mediate adaptive plasticity. Furthermore, because subcortical auditory nuclei receive cholinergic inputs (Schofield et al., 2011; Beebe and Schofield, 2021), it is possible that the plasticity that underpins auditory spatial learning takes place in the inferior colliculus or other structures that are targeted by descending projections from the auditory cortex.

## CRediT authorship contribution statement

**Fernando R. Nodal:** Writing – review & editing, Writing – original draft, Investigation, Funding acquisition, Formal analysis, Conceptualization. **Nicholas D. Leach:** Investigation. **Peter Keating:** Writing – review & editing, Software, Investigation. **Johannes C. Dahmen:** Writing – review & editing, Investigation, Formal analysis. **Dylan Zhao:** Formal analysis. **Andrew J. King:** Writing – review & editing, Writing – original draft, Resources, Project administration, Funding acquisition, Conceptualization. **Victoria M. Bajo:** Writing – review & editing, Writing – original draft, Supervision, Resources, Project administration, Methodology, Investigation, Funding acquisition, Formal analysis, Data curation, Conceptualization.

## Declaration of competing interest

The authors declare the following financial interests/personal relationships which may be considered as potential competing interests:

Andrew J King reports financial support was provided by Wellcome Trust. Victoria M Bajo reports financial support was provided by Royal National Institute for Deaf People. If there are other authors, they declare that they have no known competing financial interests or personal relationships that could have appeared to influence the work reported in this paper.

## Data Availability

Data will be made available on request.
